# Registered nurses’ experiences and preparedness for Hajj mass gatherings: an exploratory study

**DOI:** 10.1186/s12912-025-04002-y

**Published:** 2025-10-22

**Authors:** Thawab Alrabie, Michael Brown, Billiejoan Rice, Lynne Marsh

**Affiliations:** 1 School of Nursing and Midwifery, Queen’s University Belfast, Belfast, UK; 2 College of Nursing, Umm al-Qura University, Makkah al Mukarramah, Saudi Arabia

**Keywords:** Preparedness, Hajj, Mass gatherings, Nurses, Experiences

## Abstract

**Background:**

Mass gatherings such as the Hajj pose significant challenges to healthcare systems due to extreme crowd density, environmental stressors, and increased risk of medical emergencies. These events require robust preparedness and highly coordinated medical responses. However, existing literature indicates critical gaps in the readiness of registered nurses.

**Aim:**

This study explores the preparedness and experiences of Registered Nurses during the Hajj, with a focus on identifying key barriers, needs, and strategies for effective disaster preparedness and response.

**Methods:**

A qualitative exploratory design was used, involving semi-structured interviews with ten registered Nurses in Taif and Makkah cities. Data were analysed using Reflexive Thematic Analysis to explore experiences and challenges in preparedness during the Hajj.

**Findings:**

The study identified five key themes from interviews with registered nurses involved in the Hajj mass gatherings, reflecting critical aspects of their experiences in delivering care during this mass gathering event. These themes include Hajj preparedness, disaster and crisis management, patient care management, staff well-being, and communication and coordination.

**Conclusion:**

This study highlights the multifaceted challenges faced by registered nurses during the Hajj, emphasising the need for improved preparedness, culturally competent care, and coordinated response systems. It also reveals the importance of supporting both the technical and emotional dimensions of care to enhance healthcare delivery in mass gatherings.

**Clinical trial number:**

Not applicable.

**Supplementary Information:**

The online version contains supplementary material available at 10.1186/s12912-025-04002-y.

## Introduction

A mass gathering is defined as an event—whether planned or spontaneous—where the number of attendees is large enough to place considerable strain on the planning and emergency response capacities of the host community, city, or country [1]. Events such as religious pilgrimages, sporting competitions, concerts, and political rallies can overwhelm both dedicated on-site services and local healthcare systems. Most medical incidents involve minor complaints—such as dehydration, lacerations, and first aid needs—making up more than 75% of reported cases [2]. Outdoor and physically demanding events are particularly associated with environmental injuries, including sunburn, heat exhaustion, and cuts, while gatherings like music festivals, especially those attended by younger populations, are more likely to report alcohol- and drug-related emergencies [3]. These conditions pose unique logistical and clinical challenges in medical planning and emergency care delivery at such mass gathering events [4].

The Hajj pilgrimage is one of the largest and most complex annual mass gatherings globally. Each year, over two million Muslims from diverse cultural and geographical backgrounds converge on the holy city of Makkah, Kingdom of Saudi Arabia, to fulfil a religious obligation incumbent upon all physically and financially capable Muslims at least once in their lifetime [5]. The Saudi government has invested substantial resources in infrastructure, healthcare services, and emergency systems to safeguard pilgrims, reflecting its longstanding commitment to serving those undertaking the Hajj. Nevertheless, the pilgrimage also presents potential challenges in logistics, public health, and emergency management, as demonstrated by historically significant incidents such as the Mina tent fire in 1997 and the 2015 Mina stampede [6]. The congregation of large numbers of individuals within confined areas, often under difficult environmental conditions, further amplifies the potential for various forms of incidents [7].

Considering these challenges, effectively managing the Hajj requires careful planning, coordination, and rapid response. Adequate preparation helps identify at-risk populations, reduces hospital transfers, and enhances the overall quality of care [8]. Planning for mass gatherings as large and significant as Hajj involves governmental and non-governmental organisations at different levels and demands an interdisciplinary approach. Inadequate preparedness for responding to multiple incidents can have significant repercussions for healthcare, including inpatient overcrowding, treatment delays, and limited resources [9]. Therefore, comprehensive planning and careful consideration of potential and required outcomes are imperative to ensure the preparedness of healthcare systems [1].

Previous literature has underscored the multifaceted challenges of preparedness among healthcare professionals during the Hajj pilgrimage, revealing critical gaps in knowledge, training, and institutional support. Alzahrani and Kyratsis reported that more than half of emergency room nurses had never reviewed their disaster response plans, with nearly 10% unaware of such protocols altogether, indicating significant deficiencies in institutional preparedness [10]. Al-Otaibi found that EMS professionals exhibited the highest levels of preparedness, with paramedics demonstrating general disaster readiness and physicians showing a stronger grasp of Hajj-specific emergencies [11]. Similarly, Al Wathinani highlighted that older, more educated, and military-affiliated EMS personnel performed better in response, particularly when regularly engaged in workshops and hands-on training, emphasising the value of experiential learning [12].

The current study aims to explore the preparedness and response capabilities of RNs deployed during the Hajj. It seeks to explore their readiness levels, identify existing barriers to effective response, and recommend evidence-based strategies to enhance healthcare system resilience during mass gathering events.

## Methods

### Study design

This study adopted a qualitative exploratory design to investigate the experiences, roles, and perspectives of RNs in preparedness and response during the Hajj pilgrimage. The exploratory approach was selected to enable an in-depth understanding of how RNs navigate the unique challenges presented by this mass gathering event. Given the contextual complexity and the limited prior qualitative exploration of this subject in the Saudi healthcare setting, a qualitative method was deemed most appropriate to uncover rich, contextualised data [13].

### Setting

The research was conducted in two governmental hospitals: King Faisal Hospital Medical Complex in Taif city and King Abdulaziz Hospital in Makkah city. Data collection occurred over six months, from January 2024 to June 2024, aligning with the preparatory and operational phases of the Hajj. These sites were selected due to their proximity to pilgrimage routes and their roles in hosting healthcare professionals and facilities mobilised for Hajj-related medical response.

### Sampling and sample

A purposive sampling strategy was employed to ensure the inclusion of participants with direct and relevant experience of healthcare delivery during Hajj. The final sample comprised ten RNs. Eligibility criteria required participants to have at least one year of professional experience in the Hajj mass gatherings. Participants needed to be able to communicate fluently in either Arabic or English, regardless of nationality or gender, and to voluntarily consent to participate. Individuals were excluded if they lacked Hajj-related response experience, had less than one year of professional experience, were unable to communicate in Arabic or English, or declined to provide informed consent. Recruitment was facilitated through hospital nursing directors, who acted as gatekeepers by distributing information sheets and invitations to eligible staff. Additionally, recruitment leaflets were circulated within the hospitals, and a snowballing approach was used, whereby participants referred colleagues who also met the inclusion criteria. This process ensured that the nurses selected were able to provide rich and relevant insights into preparedness and response during the Hajj.

### Data collection

Data were collected through individual semi-structured interviews conducted either in Arabic or English, based on participant preference. Interviews were guided by a flexible interview schedule (see Appendix A) that included open-ended questions designed to elicit detailed accounts of participants’ roles, training, experiences, perceived challenges, and suggestions for improving response and care during the Hajj. The interview guide was developed by the researcher and reviewed by three academic experts in the nursing field to ensure content relevance and clarity. It was then piloted with five RNs, allowing the researcher to identify and refine any ambiguous or unclear questions before formal data collection. This format allowed participants to describe their experiences while also giving the researcher the flexibility to probe emerging themes in real time. All interviews were audio-recorded with participants’ permission and later transcribed verbatim for analysis. Data collection continued until data saturation was reached, defined as the point when no new codes, themes, or insights emerged from successive interviews.

### Data analysis

Thematic data analysis was conducted using Reflexive Thematic Analysis (RTA), following Braun and Clarke’s six-phase process [14]: familiarisation with the data, generating initial codes, searching for themes, reviewing themes, defining and naming themes, and producing the final report. RTA was selected for its suitability in capturing the nuanced and subjective realities of nurses working in high-pressure healthcare environments. Coding was performed inductively using NVivo software to manage and organise the data, with each transcript treated as an independent source of insight. The coding process was iterative and documented through an audit trail of notes, memos, and theme refinements. Primary coding was undertaken by the lead researcher, T.A., while preliminary findings were independently reviewed by M.B., B.J., and L.M. to ensure analytic rigour and credibility. Interview excerpts were edited to remove filler words, repeated phrases, and to address translation from Arabic to English for readability, while every effort was made to preserve participants’ original meaning and tone. Reflexivity was maintained through peer debriefing, which supported critical examination of potential biases in the interpretation process. Trustworthiness was further supported by strategies such as a transparent audit trail and member checking with participants to confirm the accuracy of interpretations.

## The findings

Out of the 42 RNs invited to take part in the study, ten RNs agreed and participated in the study, with a mix of 7 males and 3 females aged between 31 and 50 years. Their years of experience ranged from 9 to 17, and they worked in various areas, including ICU, ER, PHC, MW, and infection control. The RNs held different levels of qualifications, including bachelor’s Degrees, master’s Degrees, and Diplomas. Their participation in the Hajj ranged from 3 to 15 times, as shown in Table 1 below.

**Table 1:** RN participants

Participants	Age	Gender	Years of experience	Clinical area	Educational level	Participation No in the hajj
P1	31	Female	11	ER	Master	7
P2	45	Male	9	ICU	Master	14
P3	32	Male	17	Infection control	Bachelor	9
P4	40	Male	14	MW	Diploma	15
P5	33	Male	17	PHC	Bachelor	3
P6	35	Male	10	ER	Bachelor	5
P7	50	Female	10	MSW	Diploma	5
P8	41	Male	17	ER	Master	7
P9	38	Male	15	MW	Bachelor	9
P10	27	Female	8	SW	Bachelor	4

### Key findings

The study identified five key themes: Hajj preparedness, disaster and crisis management, patient care management, staff well-being, and communication and coordination.

#### Hajj preparedness

The key theme focused on the training and preparation of healthcare professionals before participating in Hajj. The theme highlighted the importance of pre-Hajj courses and orientation in equipping healthcare professionals. Proper preparation was identified as critical to ensuring high-quality patient care during the Hajj mass gatherings.

Two participants highlighted that governmental preparations for Hajj start well in advance, often six months before the mass gatherings. These efforts include upgrading hospital facilities with advanced medical equipment and appointing administrators to manage operations. Such proactive steps are essential for providing effective healthcare to the vast number of pilgrims. One participant stated:

Comprehensive preparations start a month ahead of Hajj, with hospitals upgraded and administrators in place to ensure efficient healthcare delivery for pilgrims. (P2)

Two participants shared that before Hajj begins, there are numerous pre-Hajj courses, particularly those centred around basic knowledge and skills such as infection control, safety and disaster management. These courses play a crucial role in preparing healthcare professionals, ensuring they are equipped with the essential expertise required to perform effectively during Hajj. One participant stated:

These courses provide essential education and training on Hajj-related topics, including health, safety, and disaster management. (P9)

The same participants explained that pre-Hajj courses not only enable healthcare professionals to perform their duties more effectively by providing essential knowledge and skills but also assist in reducing the stress associated with working in such a high-pressure environment. The training ensures participants feel prepared and confident, which minimises anxiety and allows them to focus on delivering high-quality care during the Hajj experience. One stated:

This not only helps in performing duties effectively but also aids in reducing stress during the Hajj experience. (P10)

Participants also emphasised the importance of receiving proper orientation about the equipment and procedures specific to the Hajj environment. Healthcare professionals may encounter medical equipment not commonly used in their usual hospital settings. Understanding these new tools and procedures beforehand helps to ensure that staff can use them effectively when treating pilgrims.

Orientation on the specific equipment and procedures used during Hajj is essential, as each hospital may have different tools, and being prepared helps healthcare professionals navigate new challenges more confidently. (P7)

Participants emphasized the need for a more accurate and thoughtful approach to ensure that individuals with the appropriate skills and expertise are placed in roles where they can effectively serve the pilgrims. Proper assessment of clinical competencies and relevant experience before participation is critical for maintaining high standards of care during the mass gathering. One participant stated:

Accurate workplace selection during Hajj is vital to ensure that skilled professionals are placed in roles where they can effectively support and care for the pilgrims. (P7)

Participants noted that RNs coming from outside regions, such as Tabuk, may find the Hajj environment overwhelming and challenging, despite their prior knowledge and experience. The huge volume of people and the unfamiliarity with the intense, fast-paced atmosphere can make it difficult to navigate. Even for those regularly working at the holy sites, the massive crowds can be disorienting, highlighting the complexity of the situation. One shared:

Despite their experience, RNs from outside regions may struggle with the overwhelming crowds and chaos of Hajj, as even regular staff find the environment challenging. (P4)

Participants emphasised the importance of incorporating a specialised curriculum focused on Hajj into university programs for healthcare students. Such a curriculum would help students understand the unique challenges faced during Hajj, such as the different communication styles, administrative procedures, and resource management. By providing students with knowledge tailored to these circumstances, it would better prepare them for the demands of working in this high-stress environment and motivate them to contribute meaningfully to patient care during the pilgrimage. One stated:

A small curriculum focusing on Hajj-related challenges would greatly benefit healthcare students by providing a deeper understanding of its unique aspects, preparing them for the distinct demands of working in such an environment. (P1)

Participants reflected on the valuable lessons they learned, particularly in relation to protocols, guidelines, and evidence-based practices. The experience of managing large numbers of patients, often under stressful conditions, was both challenging and rewarding. This process enhanced their ability to perform effectively in high-pressure environments and reinforced the importance of following established practices to ensure quality care.

My experience during Hajj was fulfilling, as it taught me the importance of protocols, guidelines, and evidence-based practice while performing under stress and managing large numbers of patients. (P2)

To conclude, the participants underscored that comprehensive training and preparation are essential for healthcare professionals to deliver effective care during the Hajj mass gatherings. Pre-Hajj courses, early facility upgrades, and orientation sessions on specialised equipment all contribute to better performance and reduced stress.

#### Disaster and crisis management

RN experienced challenges during emergencies such as building collapses and stampedes at Hajj mass gatherings, including emotional exhaustion, resource limitations, and communication gaps.

Five participants reported experiencing emotional and physical strain during the Hajj emergencies. The relentless pace of work left many feeling exhausted, with minimal time or opportunity for recovery between shifts. Three participants stated:

I cannot forget the disaster we had. We were really stressed during that time. We felt exhausted while trying to provide them with the best possible care. (P2)

We barely had time to catch our breath. It felt like we were constantly running from one patient to another with no break in between. (P1)

There were moments I felt like collapsing. We were so overwhelmed that even basic needs like eating or resting had to wait. (P4)

Two participants called for recognition and support for RNs during high-pressure situations. Their statements reflect a pressing need for systemic interventions to address workplace stress and ensure healthcare professionals are adequately prepared and supported in future crises. Examples of such interventions include adequate staffing, regular crisis management training, and the creation of accessible rest and recovery spaces. One participant stated:

We were all under immense stress during that time. It’s evident that more must be done to support healthcare professionals in high-pressure situations, including ensuring adequate staffing and facilitating recovery after such disasters, to better manage future crises. (P5)

One participant highlighted that the influx of patients during at Hajj can significantly impact and strain available resources, leaving healthcare systems struggling to meet demand. Participants shared that the shortage of essential supplies directly impacts the quality and timeliness of care delivery, making it challenging for healthcare professionals to address patient needs effectively during such critical periods. One said:

We had a huge number of patients during Hajj; there’s an influx of patients that strains our resources. (P6)

Additionally, two participants shared that there was a disconnect between training and execution in real-life situations. While workshops and training sessions typically cover critical topics such as communication protocols, evacuation procedures, and crisis management strategies, these preparations may fall short when faced with the unpredictability and intensity of actual emergencies, one participant noted:

Before the disaster happened, there were workshops about communication, evacuation, everything about the disaster. But when the real scenario happened, no one could use them. (P8)

One participant attributed this disconnect to the intense stress and pressure they experienced during crisis management. In highly chaotic and high-stakes situations, professionals often find it difficult to think clearly and apply their training. the participant described feeling completely frozen in the moment, unable to remember or act on their training. Another explained that, although they understood the protocols in theory, the sheer intensity of the disaster made it nearly impossible to make decisions and respond effectively.

In the midst of a disaster, the pressure and chaos can make it impossible to recall and apply the training, no matter how well it was learned. (P3)

Three participants also suggested the importance of integrating digital tools into mass gathering management systems. By leveraging technology to facilitate immediate communication, healthcare professionals improved preparedness, reduce response times, and enhance the effectiveness of interventions during emergencies. The participants stressed the growing need for modern, technology-driven solutions in managing complex and dynamic disaster situations, two stating:

We can use the application for communication. For example, if there is any issue or disaster, the application will send a notification for all healthcare professionals nearby. This will help them stay aware and prepared. Also, the application may send each role and responsibilities for each section. (P10)

During emergencies, information flow is crucial. A digital platform can help ensure that everyone receives accurate instructions immediately. (P2)

To conclude, although nurses receive formal disaster response training, a gap persists between theory and real-time practice during Hajj mass gatherings. Bridging this requires adaptive, scenario-based simulations and real-time support systems.

#### Patient care management

This theme explored the experiences of healthcare professionals in delivering patient care during the Hajj mass gatherings, highlighting the unique challenges and demands they faced in the Hajj.

Two participants explained that during the Hajj mass gatherings, there are times when challenges arise with pilgrim patients. Often, these patients required extensive attention and assessment and treatment, and in some cases, they required to be transferred to other healthcare facilities for additional care. However, some patients resisted completing their treatment, determined to continue their Hajj journey, as they see it as a once-in-a-lifetime opportunity. two participants stated:

I remember a patient from last year when I was working. He was a Pakistani male elder, around 60 or 70 years old, who came in with high blood pressure and a history from six months prior. When he arrived at the ER and we checked his vitals, after providing all necessary emergency care, the doctors gave him a preliminary diagnosis of possible cerebrovascular accident (CVA). Due to our hospital not having a CT scan, it was advised to transfer the patient to King Abdullah City, where they have the necessary facilities, specialists, and consultants. He preferred either discharge with medication or to return to his accommodations. (P7)

I came for Hajj, and nothing will stop me from completing it.’ Even after explaining the potential dangers, he refused any further care and chose to continue his pilgrimage.“(P4)

Two participants also noted that when translators were available, communication between healthcare professionals and patients became much smoother, allowing for quicker assessment and treatment. The presence of translators helped bridge the language gap, ensuring that care was timely and accurate. One participant stated:

Having a translator available makes all the difference. It allows us to understand the patient’s condition quickly and provide the care they need without unnecessary delays. (P1)

However, two participants explained that the large number of pilgrims seeking medical care sometimes creates significant pressure on healthcare professionals, making it difficult to manage patient flow efficiently. Due to the diversity of the pilgrim population, language barriers are a frequent issue, particularly when patients arrive at the ER without providing any prior medical history. One participant stated:

Such a high number of patients is due to the large number of pilgrims and language barrier issues. Sometimes patients arrive at the ER, and we do not know their history, as there is no translator available for their language, and that may delay the patient in the ER and otherwise everything is fine. (P6)

Additionally, participants highlighted the unpredictable and often overwhelming nature of healthcare provision during like Hajj mass gatherings. The sudden surges in patient numbers, due to various factors such as accidents, medical emergencies, or health-related issues, created intense pressure on healthcare professionals to act quickly and effectively. One shared:

One of the biggest challenges is the fluctuation in the number of casualties. At times, we receive thousands of patients in a single hour, and in the next hour, it might drop to just 5 or 6 patients. This inconsistency requires us to be highly adaptable and ready to handle large volumes of patients efficiently. (P10)

Patient transport in primary healthcare during Hajj sometimes presents logistical challenges for healthcare professionals. One participant explained that ambulances were sometimes not readily available, necessitating the transport of two patients in a single vehicle to higher care centres. Furthermore, most medical centres typically have only one ambulance, which may not suffice during patient surges. One stating:

Sometimes, ambulances are not available, and we must transport two patients in the same vehicle to a higher centre. Centres. Each centre typically has only one ambulance available, which may not suffice during instances of multiple patient arrivals. (P3)

To conclude, effective healthcare delivery in mass gatherings like Hajj requires careful planning, multilingual support, and culturally responsive care protocols.

#### Saff’s Well-Being

A significant theme identified from the analysis of the data was the well-being of healthcare professionals during the Hajj mass gatherings. Two participants appreciated the shared accommodations during the Hajj, saying the close arrangements helped build teamwork and stronger bonds with colleagues. This supportive environment made it easier to relax after long shifts. One stating:

We were close to each other, which made it easier to share experiences and support one another during challenging times. (P8)

However, other two participants explained that healthcare professionals often share rooms with colleagues working opposing shifts—some on night shifts and others on day shifts. This arrangement frequently disrupts rest periods, as activity and noise from those returning or preparing for shifts can disturb others who are trying to sleep. Such disruptions contribute to physical and mental exhaustion One participant stated:

Limited rest due to noise and disturbances may lead to fatigue and decreased concentration. (P9)

Similarly, two participants emphasised the lack of privacy in shared accommodation during the Hajj, particularly noting how the cramped arrangement of beds within the rooms exacerbates discomfort and limited privacy. Beds were often placed so close together that individuals have little to no personal space, making it difficult to relax or unwind after demanding shifts. One said:

The beds are so close to each other, and there’s no privacy at all. After a long and exhausting shift, having no personal space to rest or recharge makes it even harder to cope with the demands of the job. (P5)

Participants acknowledged that stress is an inevitable aspect of working during Hajj due to the high-pressure environment, large patient volumes, and unpredictable challenges. However, one participant highlighted that effective stress management strategies, such as staying organised and seeking support from colleagues, can make a significant difference. By maintaining a structured approach to tasks and fostering teamwork, healthcare professionals are better equipped to handle the demands of their work.

Stress is common in Hajj, but staying organised and seeking support from others can help manage it effectively. (P6)

Another participant shared that having strong knowledge and skills, some healthcare professionals may lose confidence during Hajj due to the high-pressure environment. The event’s fast-paced nature demands intense focus and quick decision-making, which can overwhelm even experienced individuals. The constant challenges and need for immediate action may make them feel uncertain, as they struggle to apply their expertise in such an unpredictable setting. One explained:

Sometimes, there are individuals with strong knowledge and skills who are well-educated in their field. However, when they participate in the Hajj, the unique and intense environment of the event can affect their confidence. The overwhelming circumstances require a level of attention and concentration that can make even experienced professionals feel unsure, as they are faced with situations that demand immediate and precise responses. (P4)

However, Participants reflected the deep sense of personal fulfilment healthcare professionals experience during Hajj, emphasizing how their well-being is tied to the meaningfulness of their work. One participant expressed that facilitating the spiritual journey of pilgrims—many of whom may be experiencing Hajj for the first and only time in their lives—provides a profound sense of purpose and achievement. Whether they care for a single patient or manage the needs of hundreds, the act of contributing to such an important moment in a pilgrim’s life creates a feeling of success and satisfaction. One shared:

You mean my well-being, it’s about how I feel regarding my participation. As I said before, if you feel like you are doing something meaningful for people who may be coming for the first time—and perhaps the only time—and you are helping facilitate their Hajj, you will feel successful, whether you handle one patient or a thousand patients. (P2)

Similarly, participants highlighted the strong sense of teamwork and shared purpose among the staff during Hajj. The participant emphasizes that, as Muslims, the healthcare professionals and other staff members are united in their mission to help pilgrims. They not only provide medical care but also take part in the spiritual journey of the pilgrims, with the hope that their prayers are accepted by God (Inshallah). The experience was described as collaborative and positive, with no negative aspects, suggesting that the team worked harmoniously and effectively to support the pilgrims’ needs. One explained:

It was amazing and collaborative. As Muslims, most of the staff and people there are also trying their best to help those who come to perform Hajj. We provide them with excellent care and take their prayers, hoping that God will accept them, inshallah. The collaboration was very nice, with no negatives. (P1)

To conclude, better accommodation, effective stress management, and acknowledgement of the spiritual dimension can enhance staff well-being.

#### Communication and coordination

The thematic analysis revealed a key theme focused on communication and coordination during Hajj mass gatherings. Emphasis was placed on the critical importance of clear and effective communication in ensuring smooth operations and efficient patient care throughout the Hajj mass gatherings event.

Effective communication is critical during Hajj, especially when relaying urgent medical situations. However, participants indicated that some individuals responsible for communicating such scenarios lack the necessary communication skills. This shortcoming can hinder the accurate transmission of crucial details, potentially delaying the appropriate response. One shared:

Sometimes, those tasked with communicating urgent situations lack proper communication skills, which makes it hard to convey the urgency accurately. This can delay responses and impact the care we provide. (P6)

Two participants emphasized the importance of clear and efficient communication between the Ministry of Health, the Red Crescent, and paramedics for smooth patient transfers and timely care delivery. They acknowledged that, despite the immense pressure of Hajj mass gatherings, strong coordination among these entities is evident. Enhancing such collaboration further promotes teamwork and ensures more efficient and effective patient care. One stated:

Although we face some pressure during Hajj there is a good coordination between the Ministry of Health, the Red Crescent, and paramedics would enhance coordination, ensuring faster patient transfers and better healthcare delivery. (P3)

Group WhatsApp chats have become an integral part of communication within hospitals and departments during Hajj. Participants shared that these platforms facilitate the quick sharing of information, whether it’s updates about training courses, changes in protocols, or urgent notifications during emergencies. This streamlined communication enhances coordination among team members, allowing them to respond efficiently to critical situations and ensuring the smooth delivery of care to pilgrims. One shared:

In our hospitals and departments, we use WhatsApp group chats for quick communication. They help us share updates about courses, new information, and respond swiftly during emergencies, improving coordination and overall efficiency. (P9)

Participant emphasised that integrating technology into healthcare during Hajj is crucial for enhancing patient outcomes and optimising processes. The use of telemedicine services allows healthcare professionals in the field to consult remotely with specialists from other hospitals, facilitating expert guidance and improving decision-making. This approach ensures that patients receive timely, appropriate care, particularly in complex situations, and enhances the overall efficiency and quality of healthcare delivery during the pilgrimage. One shared:

Technology is crucial in healthcare. We should prioritise its integration, especially telemedicine, to enable consultations with specialists from other hospitals and improve the quality of care provided during Hajj. (P5)

Participant highlighted the critical role of technology during the Hajj mass gathering for effective planning and response. Advanced tools that analyse data, including call volumes and the nature and location of issues, are invaluable. By utilising platforms such as dashboards to process this information, healthcare professionals can anticipate challenges and proactively prepare. This proactive approach allows for early intervention, preventing issues from escalating and ensuring a more efficient and responsive healthcare system throughout the event. One stated:

Using technology during Hajj is vital. Analysing data through tools like dashboards helps identify issues early, allowing teams to prepare and address problems before they escalate, ensuring smooth operations and effective care. (P2)

Two Participants emphasised that effective healthcare management during Hajj relies on meticulous planning and coordination, especially in improving communication between various authorities, hospitals, and organisations. Strong collaboration and efficient communication channels are essential for ensuring the optimal use of resources, timely patient care, and prompt resolution of challenges. By prioritising these efforts, the healthcare system can function cohesively, effectively meeting the demands of this large-scale event.

Our focus should be on planning and coordination, improving communication between authorities, hospitals, and organisations to ensure efficient resource use and timely patient care. (P4)

To conclude, effective communication is vital for patient safety during Hajj, underscoring the need for training, integrated systems, and stronger inter-agency coordination.

## Discussion

The participants highlighted five main themes: Hajj preparedness, disaster and crisis management, patient care management, staff well-being, and communication and coordination. themes reflect the importance of planning, rapid emergency response, and effective care delivery in a high-pressure environment. They also highlight the need to support nurses and ensure strong coordination among teams.

One of the most frequently cited strengths by participants was the provision of pre-Hajj training programs, particularly in infection control, disaster response, and safety protocols. These courses offer essential knowledge for RNs, especially those unfamiliar with the mass gathering context. However, while content breadth is often sufficient, the method of delivery lacks realism and fails to address the adaptive challenges nurses face in the dynamic environment of Hajj. Participants reported a clear disconnect between classroom-based instruction and real-life execution, particularly under the intense pressure of emergencies. These findings align with previous studies [15–17], which found that although nurses involved in emergencies or disasters had received standardised disaster response training, they frequently felt uncertain about their roles when actual emergencies occurred.

Building on this, a study conducted by Alrabie et al. further emphasised the gap between theoretical knowledge and practical preparedness. It highlighted the limitations of traditional mass gathering training and advocated for a shift toward more experiential learning. Their critique of didactic approaches and endorsement of simulation-based drills is well-supported and aligns with best practices in disaster education [18]. Consequently, the findings suggest a pressing need to transition from passive instruction to hands-on simulations that mimic the chaos, unpredictability, and urgency of Hajj conditions. High-fidelity simulations and interdisciplinary crisis drills could significantly enhance nurses’ decision-making, coordination, and emotional resilience under pressure [19].

In addition to training methods, the study also revealed issues related to limited awareness and inconsistent application of operational protocols. Despite receiving some form of orientation, several participants expressed difficulty navigating unfamiliar equipment, unclear administrative procedures, and ambiguous role expectations during deployment. This is consistent with the findings of Alzahrani and Kyratsis, who reported that ER nurses showed limited knowledge in emergency and disaster preparedness [9]. More than half had not reviewed the emergency response plan, and nearly 10% were unaware of its existence. These findings suggest a failure to ensure that frontline healthcare professionals are not only trained but also confident in the policies and procedures guiding their actions. Standardised policy briefings and role-specific orientation must therefore be reinforced throughout the deployment timeline, with digital access to updated guidelines and decision-support tools [17, 20].

Finally, despite the vital role that communication and coordination play in ensuring effective healthcare delivery during Hajj, this study reveals persistent structural and operational deficiencies that hinder optimal performance. While informal tools such as WhatsApp and dashboards have facilitated faster communication, their lack of institutional support points to a deeper issue: the absence of standardised communication frameworks. Participants noted that some staff—especially those tasked with emergency communication—lacked the necessary skills to convey critical information promptly and clearly. In high-pressure situations, this gap can lead to delayed responses, misallocated resources, and compromised patient care. Alshehri also found that participants had limited experience in disaster situations, which contributed to low confidence levels following such events [21]. To address this, targeted communication training is essential. Embedding simulation-based drills, role-playing exercises, and crisis communication workshops into pre-Hajj preparation programs can improve clarity, role understanding, and decision-making under pressure. Equipping staff with both verbal and digital communication competencies will help reduce ambiguity and enhance response efficiency [22, 23].

### Strengths and limitations

This study offers several notable strengths. First, it provides rich, firsthand insights into the experiences of RNs working during the Hajj mass gathering—an area that has received limited qualitative attention despite the complexity and scale of the event. The use of in-depth interviews allowed for the exploration of nuanced challenges and coping strategies in a culturally and logistically unique healthcare context. Second, the study identified practical, context-specific recommendations, such as the integration of digital tools, simulation-based training, and improved coordination protocols, which can inform policy and training programs not only for Hajj but for other mass gatherings globally.

However, the study also has limitations. One key limitation is that it focuses solely on RNs; excluding perspectives from other healthcare roles, such as physicians, administrators, and paramedics, may reduce the comprehensiveness of the findings. Another limitation relates to transferability, as the study examined a specific group within a unique event setting. Consequently, the findings may not be easily generalisable to other mass gatherings, such as the sporting Games. Future studies should therefore include more diverse samples and varied settings to strengthen the broader applicability of the insights.

## Conclusion

This study provides an insight into the experiences of RNs delivering healthcare during the Hajj mass gathering, revealing a complex interplay of clinical, emotional, cultural, and logistical challenges. The findings underscore the need for improved disaster preparedness, scenario-based training, culturally sensitive patient care, and structured communication and coordination mechanisms. While current systems offer foundational support through training and institutional planning, participants reported a lack of translating knowledge into effective practice under pressure. The integration of digital tools enhanced inter-agency collaboration, and the prioritisation of staff well-being emerged as key areas for improvement. Importantly, the study also highlights the intrinsic motivation and spiritual fulfilment many healthcare professionals derive from participating in Hajj, suggesting that future interventions should support both the technical and emotional dimensions of care. These insights not only inform strategies for strengthening Hajj-specific healthcare delivery but also contribute to broader discussions on managing health systems in mass gatherings globally. Further research involving diverse professional groups is recommended to build a more comprehensive understanding and support evidence-based policy and practice.

## Supplementary Information

Below is the link to the electronic supplementary material.

Supplementary Material 1

Supplementary Material 2

## Data Availability

No, I do not have any research data outside the submitted manuscript file.
