# Supplementary material for: Registered nurses’ experiences and preparedness for Hajj mass gatherings: an exploratory study
Source: BMC Nurs. 2025 Oct 22;24:1309. doi: 10.1186/s12912-025-04002-y (PMC12542092; doi:10.1186/s12912-025-04002-y)
Supplement: Supplementary file 2 — Supplementary Material 2 [file 12912_2025_4002_MOESM2_ESM.docx]

**
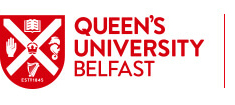
Appendix A: Semi‐structured interview questions guide**

***Opening Statements:***

Hello and good morning. My name is Thawab Alrabie, and I am conducting a research study to explore the views and experiences of registered nurses who provide care during the Hajj pilgrimage. Thank you for agreeing to participate. With your permission, I would like to begin the interview.

***Initial Questions:***

To start with the interview, I would like to ask some questions relevant to your work:

1. *How long have you qualified as a RN?*
2. *What is your job title in the clinical area that you work in?*
3. *How long have you been working in this clinical area?*
4. *Have you been working a different role in other clinical areas?*
5. *What do you like and what is most enjoyable about working in the clinical area?*

***Interview Questions:***

1. What are your key responsibilities during the Hajj, and how do they differ from your regular duties outside of the pilgrimage period?
2. What unique clinical, logistical, or cultural challenges do you face when providing patient care during Hajj?
3. How do you and your team prioritize care and make decisions in the high-pressure, mass-gathering environment of Hajj?
4. How prepared do you feel to respond to disaster situations or large-scale emergencies during Hajj?
5. How effective is the current interprofessional collaboration and communication during Hajj-related incidents, and what improvements would you recommend?
6. What resources, training, or support systems have prepared you for your role during Hajj, and what additional education or tools do you feel are needed?
7. Can you share a particularly memorable or meaningful experience from Hajj that illustrates the emotional or professional impact of your role?
